# Peptide Ligands Incorporated into the Threefold Spike Capsid Domain to Re-Direct Gene Transduction of AAV8 and AAV9 In Vivo

**DOI:** 10.1371/journal.pone.0023101

**Published:** 2011-08-05

**Authors:** Stefan Michelfelder, Karl Varadi, Christina Raupp, Agnes Hunger, Jakob Körbelin, Christiane Pahrmann, Sonja Schrepfer, Oliver J. Müller, Jürgen A. Kleinschmidt, Martin Trepel

**Affiliations:** 1 Department of Oncology and Hematology, Hubertus Wald Cancer Center, University Medical Center Hamburg-Eppendorf, Hamburg, Germany; 2 Transplant and Stem Cell Immunobiology Lab, University Medical Center Hamburg-Eppendorf, Hamburg, Germany; 3 Internal Medicine III, University Medical Center Heidelberg, Im Neuenheimer Feld 10, Heidelberg, Germany; 4 Deutsches Krebsforschungszentrum, Im Neuenheimer Feld 242, Heidelberg, Germany; Emory University School of Medicine, United States of America

## Abstract

Efficiency and specificity of viral vectors are vital issues in gene therapy. Insertion of peptide ligands into the adeno-associated viral (AAV) capsid at receptor binding sites can re-target AAV2-derived vectors to alternative cell types. Also, the use of serotypes AAV8 and -9 is more efficient than AAV2 for gene transfer to certain tissues in vivo. Consequently, re-targeting of these serotypes by ligand insertion could be a promising approach but has not been explored so far. Here, we generated AAV8 and -9 vectors displaying peptides in the threefold spike capsid domain. These peptides had been selected from peptide libraries displayed on capsids of AAV serotype 2 to optimize systemic gene delivery to murine lung tissue and to breast cancer tissue in PymT transgenic mice (PymT). Such peptide insertions at position 590 of the AAV8 capsid and position 589 of the AAV9 capsid changed the transduction properties of both serotypes. However, both peptides inserted in AAV8 did not result in the same changes of tissue tropism as they did in AAV2. While the AAV2 peptides selected on murine lung tissue did not alter tropism of serotypes 8 and -9, insertion of the AAV2-derived peptide selected on breast cancer tissue augmented tumor gene delivery in both serotypes. Further, this peptide mediated a strong but unspecific in vivo gene transfer for AAV8 and abrogated transduction of various control tissues for AAV9. Our findings indicate that peptide insertion into defined sites of AAV8 and -9 capsids can change and improve their efficiency and specificity compared to their wild type variants and to AAV2, making these insertion sites attractive for the generation of novel targeted vectors in these serotypes.

## Introduction

Systemic vector administration would be the most appropriate delivery pathway in many, if not most, potential gene therapy applications, especially in disseminated disease when local administration is not feasible. Adeno-associated viruses (AAV) provide a well established platform for the development of safe and efficient vector systems [1], [2], [3], [4]. Among the various serotypes, AAV8 and -9 have features making them particularly attractive as vectors. Upon systemic administration, AAV8 and -9 cross vascular endothelial barriers more efficiently than other serotypes, resulting in efficient gene delivery to hepatocytes, cardiac and skeletal muscle cells [5], [6], [7]. AAV9 can even efficiently deliver genes to various cell-types of the central nervous system [8] and has the unique property to cross the blood-brain barrier (BBB) [9], [10]. Another advantage over AAV2 vectors is an only moderate sero-prevalence of antibodies against AAV8 and -9 among the human population, potentially facilitating the immune escape of vectors *in vivo* [11]. All of these properties may contribute to the fact that AAV8 and -9 deliver genes to a wider range of tissues and with higher efficiency compared to other AAV serotypes [6], [7], [12], [13]. This may, however, also account for some major drawbacks regarding their utility as therapeutic vectors, because their broad and efficient tissue tropism may result in un-intended transduction of various tissues. In addition, the inefficient transduction of certain tissues may also limit their utility for distinct potential applications. Therefore, the development of cell- or tissue-targeted AAV8 and -9 vector capsids appears to be an important goal.

Receptor-directed targeting of AAV has been achieved most often by genetic insertions into the *cap* gene of AAV2, resulting in display of small peptide ligands (up to 34 amino acids) [4], [14]. Suitable peptides binding to cellular receptors can be identified by screening combinatorial peptide libraries displayed on bacteriophage [15], [16], [17], [18], [19] which subsequently may be introduced into the viral capsid [20], [21], [22], [23], [24], [25], [26], [27]. Alternatively, AAV peptide libraries in which random peptides are displayed within the viral capsid can be used for this purpose [28], [29], [30]. Screening random AAV peptide libraries has several advantages over insertion of ligands selected by phage display since the selection of the suitable peptide takes into account the structural constraints of the viral capsid and therefore will reveal only capsid variants that are compatible with virus attachment and post-entry processing within the target cell. Such AAV peptide libraries have been used to target a variety of cell types *in vitro* [20], [21], [22], [28], [29], [30], [31], [32]. Recently, this challenging approach has been translated into *in vivo* applications, yielding capsid variants with improved tropism towards murine lung [32], breast cancer [32], or cardiac tissue [33].

The capsid region surrounding amino acid R588 (VP numbering) has been used most often for the incorporation of peptide ligands into AAV2 for several reasons. Such peptide insertions are compatible with capsid assembly. Furthermore, the targeting peptide is presented 60 times near the top of protruding capsid domains within the threefold spike region, facilitating the interaction with potential cell surface structures [20], [32]. Since AAV2 attachment to its primary cellular receptor heparan sulfate proteoglycan (HSPG) is mediated by a cluster of basic amino acid residues within this capsid region surrounding position 588 [34], manipulating this region including insertion of peptides results in abrogation of HSPG binding. *In vivo*, this results in de-targeting AAV2 from its main target tissue, the liver [32], [33], [35]. Furthermore, such modifications may mediate escape from neutralizing antibodies against the AAV2 capsid [36].

The aim of this study was to re-target AAV8 and -9-based vectors to alternative tissues *in vivo* by genetic insertion of peptide ligands into domains putatively involved in receptor binding. The topology of AAV8 is based on the same structure previously described for AAV2 and comprises a characteristic eight stranded ß-barrel with large interstrand loop domains. Significant differences between AAV8 and -2 are located in protruding surface residues adjacent to the three axes of symmetry [37]. Capsid swapping analysis between AAV2 and -8 identified interstrand loop domains exposed within the threefold proximal peak (mainly loop IV domain, subloops 1–4) as the critical structural determinant for AAV8 transduction [38]. The 37/67-kDa laminin receptor (lamR) has been identified as a cellular receptor for AAV8 and -9 and residues 491–547 within the protrusions that surround the threefold axis as well as 593–623 near this axis [39] may be the respective capsid binding sites for AAV8. Koerber *et al.* inserted a hexahistidine (His_6_) at position 590 that allowed purification of AAV8 vectors *via* immobilized metal affinity chromatography (IMAC). However, *in vivo* gene transduction of such mutant AAV8 capsids was unchanged compared to its wild type origin [40].

Here we used the site adjacent to the threefold-spike domain in the capsid of AAV serotypes 8 and -9 and explored the effects of inserting peptides selected by screening AAV2 peptide libraries in putatively equivalent sites of AAV8 and -9. We show that introduction of such peptides can alter the wild type tropism of AAV8 and -9 and enhance gene delivery to certain tissues, some being and some not being the originally intended target. These effects were serotype-dependent. Re-direction of AAV8 and -9 vectors by tumor-homing peptides selected from AAV2 libraries improved gene transfer efficiency to breast cancer tissue even beyond the level of the equivalent re-directed AAV2 variant, while, particularly in AAV9, gene expression in various control tissues was diminished. Our findings demonstrate that re-direction of the tropism of AAV serotypes other than AAV2 can change and partially improve efficiency and specificity of the vectors even when the peptides used therefore were initially selected from AAV2 libraries. In addition, the tumor-targeted AAV9 vector presented here may be used in pre-clinical studies to improve breast cancer transduction *in vivo*.

## Materials and Methods

### Plasmids

To generate an AAV8 backbone plasmid with a peptide insertion site, *Sfi*I binding sites had to be inserted into the AAV8 cap gene of p5E18-VD2/8 [41]. The *Sfi*I restriction sites within the sequence of AAV8 were established by synthesizing a 743 bp AAV8 cap gene segment (Geneart, Regensburg, Germany). The restriction enzymes Xcm*I* and Eco47*III* were used to insert the sequence containing the *Sfi*I sites into p5E18-VD2/8 backbone (kindly provided by J. Wilson, University of Pennsylvania, Philadelphia, USA), resulting in p5E18-VD2/8-*Sfi*I.

For generation of the AAV9 plasmid suitable for oligonucleotide insertion, a 975 bp-fragment of the AAV9 *cap* ORF containing two *Sfi*I restriction sites was synthesized (Geneart, Regensburg, Germany). The two *Sfi*I sites were separated by two adenines to allow for directed cloning of oligonucleotides directly downstream of the trinucleotide encoding amino acid position 589 into the *cap* gene of AAV9. The analogous AAV9 wild type fragment within p5E18-VD2/9 [41] (kindly provided by J. Wilson, University of Pennsylvania, Philadelphia, USA) was replaced with the *Sfi*I-modified version by *BsiW*I/*Xcm*I-excision and termed p5E18-VD2/9-*Sfi*I1759. The oligonucleotides encoding the peptides VRRPRFW, ESGLSQS, PRSTSDP were amplified from pMTpXX2-187 plasmids [31], digested by *Bgl*I and cloned into p5E18-VD2/9-*Sfi*I1759 or p5E18-VD2/8-*Sfi*I.

### Cell culture, vector production and titering

Human embryonic kidney (HEK) 293T cells (kindly provided by David Baltimore, California, Institute of Technology, Pasadena, CA) were grown in Dulbecco's Modified Eagle's medium (DMEM; Invitrogen, Carlsbad, CA) supplemented with 1% penicillin/streptomycin solution (Invitrogen) and 10% fetal calf serum (Biochrom, Berlin, Germany). Recombinant AAV (rAAV) vectors were produced by Polyfect transfection (Qiagen) cells of plasmid DNA into HEK 293T (see below). Four days after transfection, cells were harvested, lysed and vectors were purified by iodixanol density gradient ultracentrifugation and stored at −80°C. For quantification of vector stocks, genomic titers were determined by quantitative real-time PCR using the LightCycler system (Roche Diagnostics, Mannheim, Germany), as described previously [42]. For transfections, we used pXX6 as adenoviral-helper plasmid [43], the luciferase-reporter gene pUF2-CMV-luc [28], and a plasmid encoding the AAV capsid of interest. Plasmids encoding the respective AAV capsid mutants and wild type controls were pXX2 [43], and modified pXX2-187 for AAV2 displaying peptides [31], p5E18-VD2/8 and p5E18-VD2/8-SfiI for AAV8 and p5E18-VD2/9 and modified p5E18-VD2/9–*Sfi*I1759 for AAV9, as described above.

### Animals and detection of luciferase transgene expression and vector distribution in vivo

Polyoma middle T transgenic mice (strain FVB/N-TgN(MMTVPyVT)634-Mul) were purchased from Jackson Laboratory (Bar Harbor, ME). Genotyping and tumor staging was performed as described previously [32]. All experiments involving animals were conducted in accordance with the Guide for the Care and Use of Laboratory Animals published by the US National Institutes of Health (NIH Publication No. 85-23, revised 1996) and the German Animal Protection Code. The protocol was approved by the responsible local authority (“Amt für Gesundheit und Verbraucherschutz, Hansestadt Hamburg”, permission number 42-09). All experiments were performed under deep anesthesia as described below, and all efforts were made to minimize suffering. For the analysis of luciferase gene transduction, 8–12 weeks-old female animals were anesthetized by intraperitoneal injection of 100 mg/kg body weight 10% ketamine hydrochloride (115.34 mg/ml; Essex, Munich, Germany) and 5 mg/kg body weight 2% xylazine hydrochloride (23.32 mg/ml; Bayer, Leverkusen, Germany). AAV vectors were injected intravenously into the tail vein at a dose of 2×10^10^ vector genomes (vg) per mouse in a total volume of 200 µl phosphate-buffered saline (n = 3 animals per injected AAV clone). At days 14 and 28, mice were anesthetized with 2% isoflurane and oxygen. Luciferase expression was analysed using a Xenogen IVIS200 imaging system (Caliper Lifesciences) after intraperitoneal injection of 100 µl luciferin substrate (150 mg/kg, Xenogen). Representative *in vivo* bioluminescence transgene expression images were taken when luminescence in relative light units (photons/sec/cm^2^) reached the highest intensity. Subsequently, the tissues were removed, snap frozen in liquid nitrogen, and stored at −80°C. Homogenization was done in reporter lysis buffer (RLB, Promega, Madison, WI) followed by determination of the luciferase reporter gene activity in a luminometer (Mithras LB 940, Berthold Technologies, Bad Wildbad, Germany) using Promega's luciferase assay according to the manufacturer's instructions. Values were normalized to protein levels in each sample determined by the Roti Nanoquant Protein assay (Roth, Karlsruhe, Germany). For quantification of vector genome copy number in various tissues, total tissue DNA was extracted using the DNeasy tissue kit (Qiagen) and quantified using a spectral photometer Nanodrop ND-2000C (Peqlab, Erlangen, Germany). Analysis of AAV vector DNA in tissues was performed using CMV-specific primers by quantitative real-time PCR as described above, using 100 ng of template DNA.

### Statistics

All data were expressed as mean ± SEM. Statistical analysis was performed using the GraphPad Prism program 3.0 (GraphPad Software, San Diego, CA). Data were analyzed by an unpaired students-t test. p values<0.05 were considered significant.

## Results

### Generation of AAV8 and -9 vectors displaying peptides in capsid domains forming exposed spikes on the capsid surface

**Figure 1:**
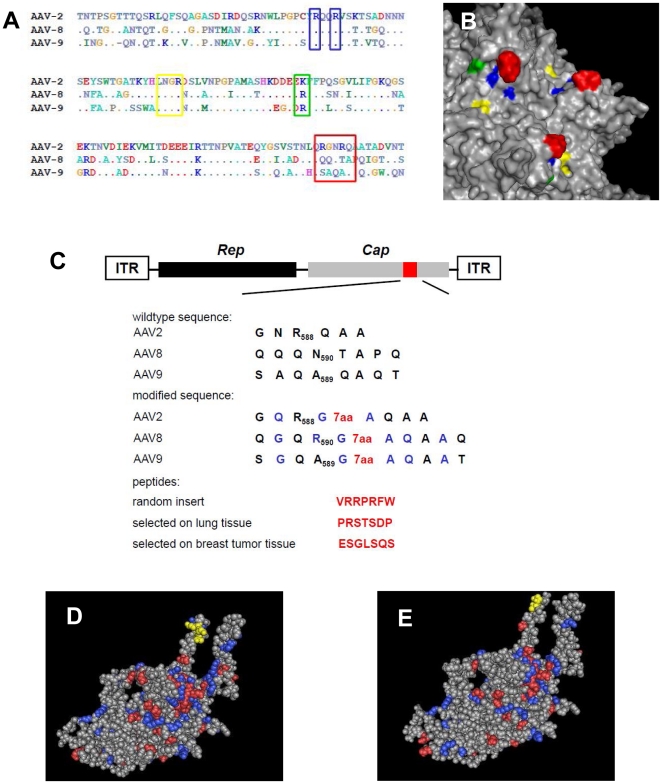
Design of novel peptide insertion sites in capsid regions adjacent to the threefold-spike in serotypes AAV8 and AAV9. **A:** Sequence alignment of surface exposed capsid domains encoded by the *cap* gene of the AAV serotypes 2, 8, and 9. Parts of the heparin binding domain 484-RQQR-487 and the integrin binding motif 511-NGR-513 are strongly conserved among the three serotypes. While AAV8 and -9 both contain an R at position 532, marked differences in the sequences are apparent at positions 585–588. Domains highlighted in B are tagged by colored rectangles. Alignment of the serotype sequences was carried out using BioEdit Sequence Alignment Editor Software. **B:** Capsid domains of AAV2 known to be involved in receptor binding are highlighted in blue (R484; R487), yellow (511-NGR-513), green (K532), and red (R585; R588). Surface rendering and mapping of the threefold-spike region was performed using PyMOL with the crystal structure of AAV2 supplied as template (PDB ID: 1lp3 [50]). **C:** Design of AAV8 and -9 constructs for the incorporation of oligonucleotides encoding targeting peptides into the *cap* gene. The amino acid sequence of the *cap* gene for each corresponding serotype (single letter amino acid code) is depicted in black letters; differences compared to the respective wild type sequence are shown in blue letters. Red indicates seven additional amino acid residues from the insertion of oligonucleotides encoding a known peptide ligand for the re-direction of AAV serotypes. **D:** Model of the VP-3 capsid protein of AAV2 and localization of R588 and **E:** model of the VP-3 protein of AAV8 capsid and localization of R590. The potential peptide insertion site is depicted in yellow. Basic amino acids are depicted in red, acidic amino acids in blue. The VP-3 model of AAV2 and AAV8 was generated using Cn3D with coordinates PDB ID: 1lp3 for AAV2 [50] and PDB ID: 2QA0 for AAV8 [37] serving as template.

To develop targeted AAV vectors on the basis of serotypes 8 and 9, we identified potential capsid regions amenable for the insertion of peptides that may re-direct the natural viral tropism. Since AAV2, -8, and -9 proteins share about 83% of their capsid amino acid sequences [37], [44], we reasoned that sequence alignment would identify domains potentially mediating the differential tropism of these three serotypes. Comparison of the threefold spike regions of the AAV2 VP3 subunit comprising the HSPG binding domain in AAV2 with corresponding VP sequences of serotypes 8 and 9 revealed highly conserved regions (R484/R488), or regions that differ by one amino acid (K532) without affecting the net charge. However, a strong variability in capsid regions surrounding R585/R588 suggested that this domain could contribute to the tropism of these serotypes (Figure 1A–B). To generate backbone plasmids for the insertion of oligonucleotides coding for potential targeting peptides, we modified the wild type AAV8 capsid sequence from QQQN_590_-TAPQ to QGQR_590_-G-Insert-AQAAQ by *in vitro* gene synthesis and the wild type AAV9 capsid sequence from AQAQ_590_- AQTTGW to GQA_589_-G-Insert-AQAATGW by *in vitro* synthesis of a part of the *cap* gene (Figure 1C). The resulting two insertion-backbone plasmids each contained two *Sfi*I restriction sites, separated by a stuffer allowing the directed insertion of oligonucleotides encoding a potential targeting peptide. To investigate whether the insertion of peptides can alter the tropism of AAV8 and AAV9-derived vectors, we engineered a set of vector variants displaying peptides selected from AAV2 peptide libraries [32]. We produced recombinant AAV2, -8, and -9 vectors displaying the peptides VRRPRFW (random control), PRSTSDP (selected for lung transduction), and ESGLSQS (selected for breast and breast tumor tissue transduction) (Figure 1C). Structural modeling of position R590 in AAV8 vectors indicated that the peptide is positioned within the threefold spike region, similar to the peptides inserted at position R588 within the AAV2 capsid (Figure 1D–E). Genomic titers of all capsid variants ranged between 8×10^10^ to 7×10^11^ vg/ml (Table 1). These data suggest that peptide insertions in these capsid domains do not impair genome packaging and capsid assembly.

**Table 1:** Titers of recombinant AAV vectors.

luciferase vector	genomic titer*[vector genomes/ml]
AAV2 wild type	9.75×10^10^
AAV8 wild type	1.63×10^11^
AAV9 wild type	5.35×10^11^
AAV2-VRRPRFW	7.17×10^10^
AAV8-VRRPRFW	2.30×10^11^
AAV9-VRRPRFW	1.87×10^11^
AAV2-PRSTSDP	1.28×10^11^
AAV8-PRSTSDP	3.63×10^11^
AAV9-PRSTSDP	6.03×10^11^
AAV2-ESGLSQS	1.29×10^11^
AAV8-ESGLSQS	5.30×10^11^
AAV9-ESGLSQS	2.46×10^11^

*vector genomes of viral stocks were determined by quantitative real-time PCR.

### Insertion of targeting peptides changes the tropism of AAV8 and AAV9 vectors in vivo

**Figure 2:**
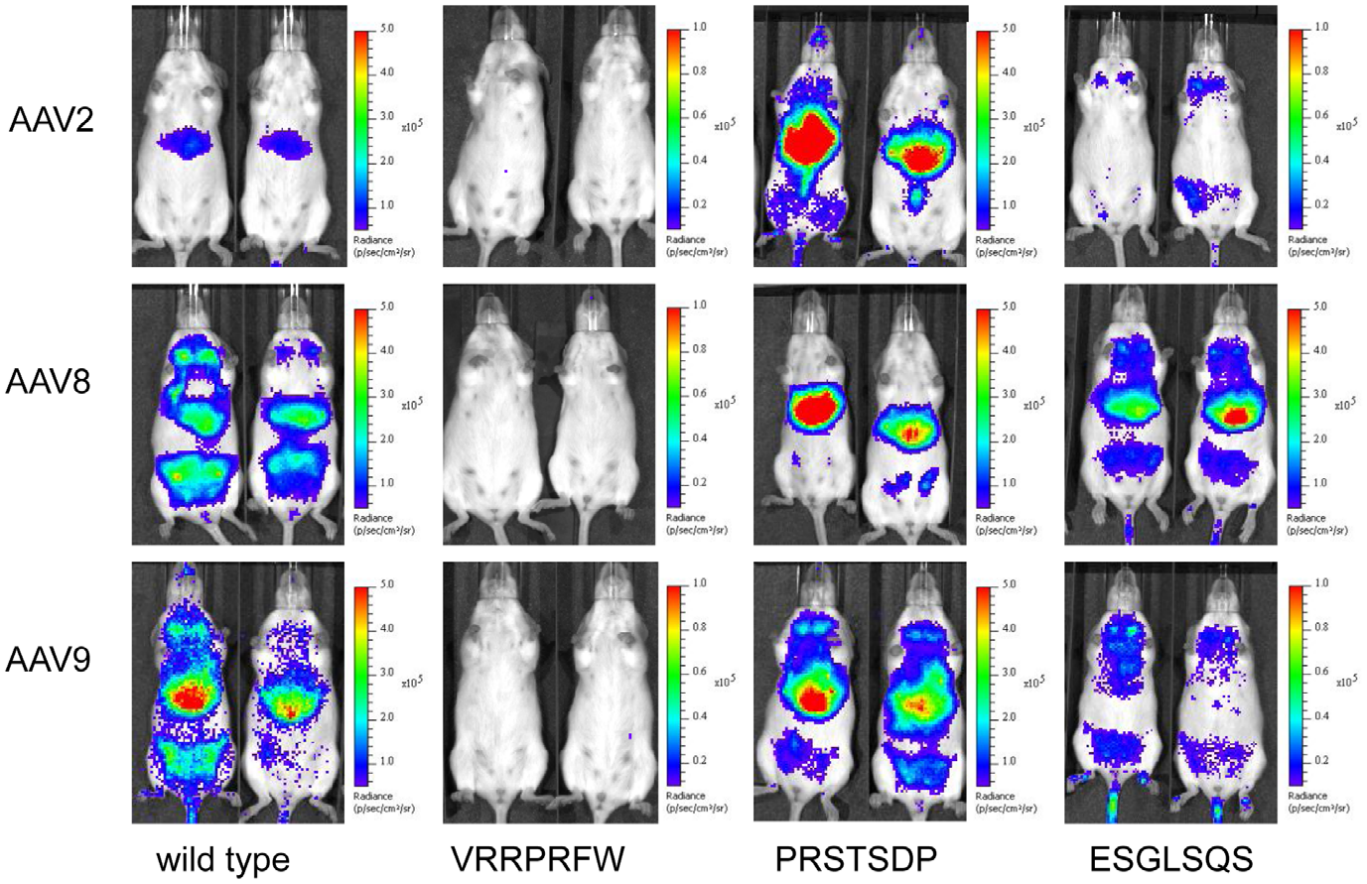
Bioluminescence imaging of gene transduction by vectors derived from AAV2, -8 and -9 displaying peptide ligands. In vivo bioluminescence imaging of transgene expression in FVB mice injected intravenously with rAAV-luciferase vectors harboring wild type capsid or capsids displaying a targeting peptide selected in the structural context of AAV2. Images were taken 28 days after vector injection, when whole animal bioluminescence intensities (BLI) reached peak values after injection of luciferase substrate. BLI ranges from 10^5^–10^8^ relative light units per animal (photons/sec/cm^2^).

To investigate the *in vivo* tropism of the vector variants described above, we analyzed luciferase activity in FVB wild type mice 28 days after low vector dose tail vein injections (2×10^10^ vg/animal) by bioluminescence imaging (Figure 2). The insertion of a control peptide (VRRPRFW) into the capsid of AAV2, -8 and -9 resulted in transduction-deficient vectors. Insertion of a peptide ligand used to augment gene transfer of AAV2 in murine lung, liver and cardiac tissue (PRSTSDP) slightly decreased transgene expression of AAV8 in the skeletal muscle. For AAV9, the peptide did not markedly alter the tropism (Figure 2). As expected, insertion of ESGLSQS into AAV2 capsids resulted in transduction of the mammary tissue, while the transgene-mediated liver luminescence disappeared. Introduced into the AAV9 capsid, the ESGLSQS peptide de-targeted expression mainly from the liver, adopting a tropism similar to AAV2-ESGLSQS.

**Figure 3:**
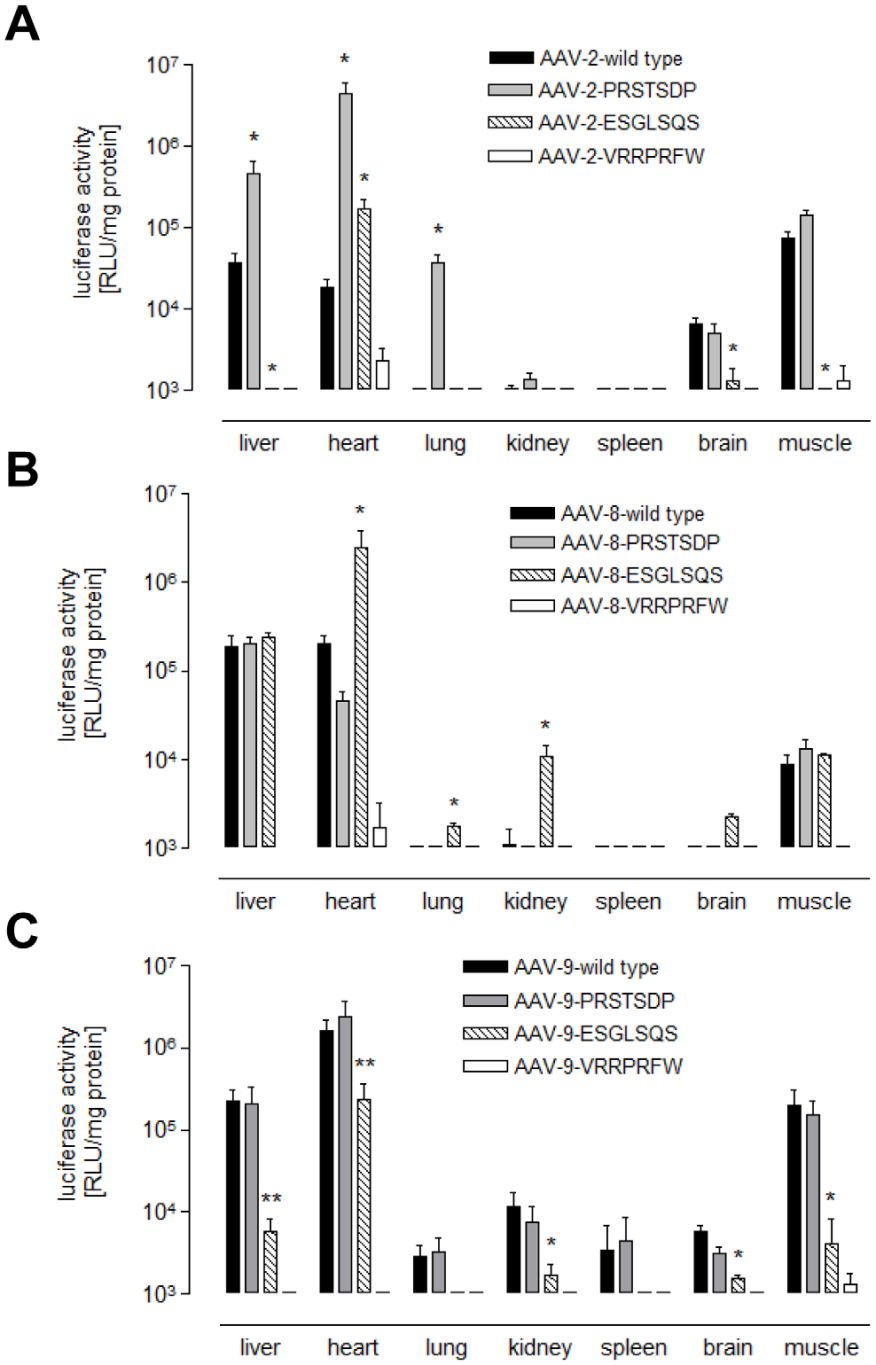
Ex vivo determination of transgene expression after systemic administration of AAV2, AAV8 and AAV9 vectors displaying peptide ligands. Luciferase gene expression in various tissues was analyzed 28 days after intravenous injection of rAAV vectors into FVB mice. Capsid insertion of the random VRRPRFW peptide did not result in transgene expression in any organ, while the lung-directed peptide PRSTSDP and the breast tissue-directed peptide ESGLSQS resulted in specific changes of tissue tropism. **A** shows results for AAV2-, **B** for AAV8-, and **C** for AAV9-injected animals. Values below 10^3^ RLU/mg protein are not shown because this indicates the threshold beyond which luciferase expression data could be reproducibly delineated from background signal. * = p<0.05; ** = p<0.01; *** = p<0.001 capsid displaying peptide versus respective wild type control (n = 3 animals).

**Figure 4:**
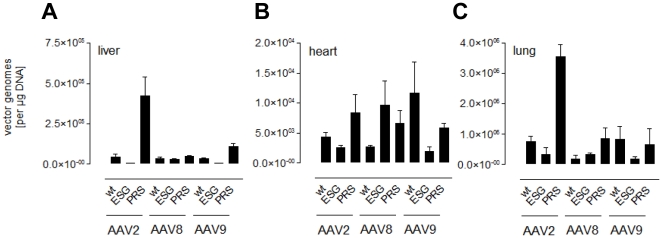
Biodistribution analysis of tropism-modified vectors. Vector genome copy numbers in various tissues were quantified by quantitative real-time PCR using CMV-promoter-specific primers. Tissues were harvested 28 days after intravenous injection of capsid-modified or wild type vectors, respectively. Recovered vector genome numbers are shown for **A** liver, **B** cardiac, and **C** lung tissue. (n = 3 animals per group).

We additionally determined the luciferase activity from crude tissue lysates. Consistent with the *in vivo* imaging results, control vectors displaying a randomly chosen peptide (VRRPRFW) did not mediate gene expression above background in all serotypes (Figure 3). As expected, insertion of the lung-directed peptide PRSTSDP significantly enhanced AAV2-mediated transduction not only of the lung, but also of heart and liver (Figure 3A). For heart transduction, AAV2 PRSTSDP was even more efficient than the most efficient AAV serotype for cardiac transduction, AAV9 (AAV2-PRSTSDP: 4.6×10^6^ RLU/mg protein vs. AAV9 wild type: 1.6×10^6^ RLU/mg protein; Figure 3A+C). When introduced into AAV8, PRSTSDP lead to a slight but not significant decrease in heart transduction compared to wild type AAV8, while insertion of PRSTSDP had no influence on AAV9 transduction (Figure 3B+C). Even though selected for optimized lung transduction, AAV2-PRSTSDP was the most efficient vector for lung transduction in the three-fold comparison. Insertion of the ESGLSQS peptide into AAV8 significantly improved gene delivery to the lung (3.42-fold), kidney (9.88-fold), brain (9.08-fold), and particularly to the heart (12.15-fold), which was even more efficient than wild type AAV9 (AAV8-ESGLSQS 2.4×10^6^ RLU/mg protein). In contrast, insertion of ESGLSQS into AAV9 capsids significantly decreased AAV9 gene transfer to almost all control tissues including the liver, the heart, and the skeletal muscle. These findings were supported by biodistribution analysis of vector genome copy numbers by quantitative real-time PCR (Figure 4). For AAV2-PRSTSDP, a high copy number was recovered in the liver (Figure 4A), in the cardiac muscle (Figure 4B). In the comparison, the highest copy number was recovered in the lung of mice injected with AAV2-PRSTSDP (Figure 4C). For AAV8-ESGLSQS, a higher amount of vector DNA was detected within cardiac and lung tissue compared to AAV8 wild type capsids (Figure 4B, C), while inconsistently to the data we obtained by expression analysis, the highest copy number we recovered in the cardiac muscle was from AAV9 (Figure 4B). The amount of vector DNA copies in mice injected with AAV9-ESGLSQS were about ∼21-fold lower in the liver and about ∼6-fold (for AAV9) lower in the cardiac muscle compared to AAV9 wild type (Figure 4A, B), confirming the de-targeting in these tissues mediated by the peptide selected on breast tumor tissue.

### Insertion of breast cancer targeting peptide ligand re-directs gene transfer of AAV8 and AAV9 to polyoma middle T-induced breast cancer tissue

**Figure 5:**
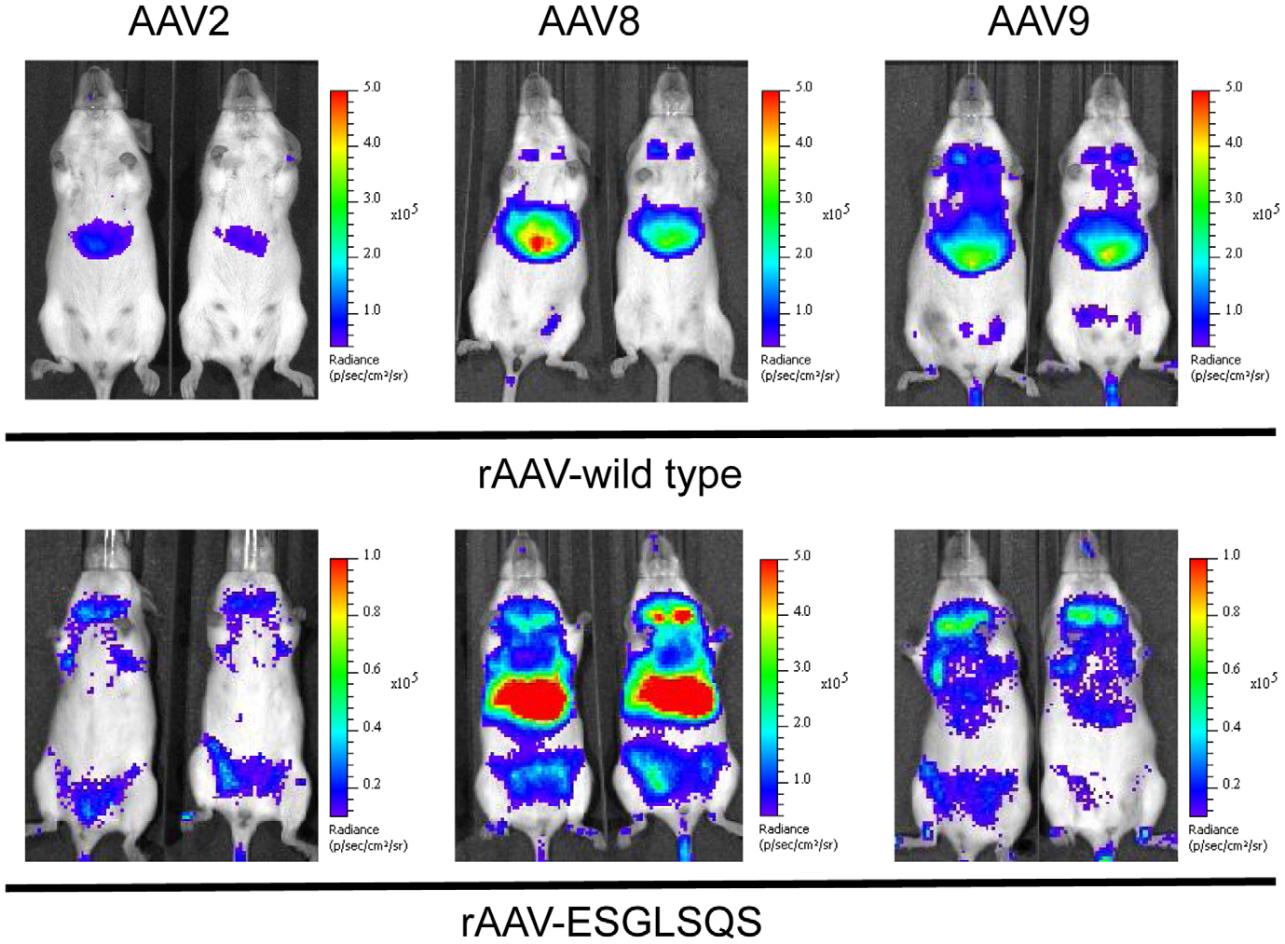
In vivo bioluminescence of gene expression in breast cancer tissue of PymT-transgenic mice. Luciferase vectors derived from AAV2, -8 and -9 displaying breast cancer-directed peptides (ESGLSQS) and respective wild type capsid vectors were injected intravenously into tumor-bearing mice (n = 3 per group). Images were taken 14 days after vector injection. BLI ranged from 10^5^–10^8^ relative light units per animal (photons/sec/cm^2^).

**Figure 6:**
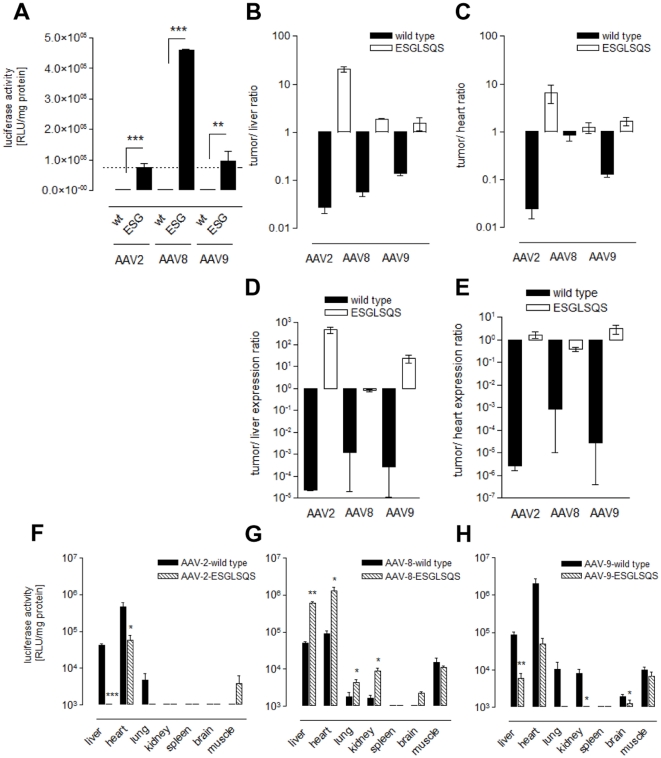
Breast cancer-directed gene delivery by AAV capsids displaying ESGLSQS depends on the underlying capsid serotype. **A:** Luciferase activities in PymT-induced breast cancer tissue were determined as relative light units (RLU) per mg protein 14 days after injection of AAV2, -8 and -9 displaying breast cancer-directed peptides (ESGLSQS) or respective wild type capsid variants (n = 3). **B and C:** the amount of vector genomes recovered in tumor, liver and cardiac tissue was analyzed by quantitative real-time PCR using the tissue samples as described in A (n = 3 animals per group). **B:** ratio of vector genome copy numbers tumor/liver (n = 3 animals per group); **C:** ratio of vector genome copy numbers tumor/heart. **D and E:** ratios of gene transduction using AAV vectors of serotypes 2, 8 and 9 with insertion of the ESGLSQS peptide and respective wild type variants. Values were calculated as **D:** tumor/liver expression ratios or **E:** tumor/heart expression ratios, respectively. **F, G and H:** Luciferase activities in various tissues were detected as relative light units (RLU) per mg protein for AAV-2 (**F**) for AAV-8 (**G**) and AAV-9 (**H**) displaying breast cancer-directed peptides (ESGLSQS) or respective AAV wild type capsid variants (n = 3 animals per group).

Since the primary target the ESGLSQS peptide was selected for was breast cancer tissue, gene transfer of ESGLSQS vectors of all three serotypes was analyzed in polyoma middle T (PymT) transgenic mice bearing multifocal breast tumors. Transgene expression was determined in tumor tissue 14 days after intravenous injection of luciferase vectors. While, as in FVB mice without tumors, AAV8-ESGLSQS vectors mediated a strong liver bioluminescence, apparent re-direction of transgene expression to breast cancer tissue was observed with AAV2-ESGLSQS and AAV9-ESGLSQS vectors, being more efficient for AAV9-ESGLSQS than in AAV2-ESGLSQS. Tumor transgene expression was not detected after injection of wild type variants (Figure 5). These findings where confirmed by single organ lysate expression analysis. The insertion of ESGLSQS resulted in strong gene transduction of breast cancer tissue in all AAV serotype mutants compared to their wild type origins (Figure 6A) and even more efficient for AAV8-ESGLSQS (4.6×10^5^RLU/mg protein) and AAV-9-ESGLSQS (9.7×10^4^ RLU/mg protein) than for the “original” AAV2-ESGLSQS (7.6×10^4^ RLU/mg protein). These findings were supported by biodistribution analysis of vector genome copy numbers in different tissues. The ratio of vector genomes recovered in the tumor tissue in relation to the amount of vector genomes recovered from the liver (Figure 6 B), respectively from the cardiac tissue (Figure 6C) revealed favorable ratios for all three serotypes displaying ESGLSQS compared to their respective wild type capsids, supporting vector homing to PymT induced breast tumor tissue. Interestingly, tumor/liver expression ratio was most favorable with AAV2-ESGLSQS (Figure 6D), while the tumor/heart expression ratio was slightly increased with AAV9-ESGLSQS vectors (Figure 6E). As expected, single organ analysis of gene expression in various tissues revealed strong but unspecific gene transduction mediated by AAV8-ESGLSQS (Figure 6G). For AAV9-ESGLSQS, we observed a strong de-targeting of gene expression in liver, heart, kidney and brain compared to wild type AAV9 (Figure 6H). However, gene transduction was not entirely specific to tumor tissue and was also detected in the heart as was observed for AAV2-derived vectors (Figure 6F) [32].

Taken together, our data suggest superior tumor/liver and tumor/heart expression ratios for AAV2-ESGLSQS vectors, indicating preferential targeting of breast tumor tissue by AAV2-ESGLSQS. The ratio of tumor/liver expression is more favorable for AAV2- compared to AAV9-ESGLSQS, because it seems to confer a more pronounced de-targeting of gene expression in the liver (Figure 6F). In turn, the expression ratio of tumor/heart is more tumor-specific for AAV9- than for AAV2-ESGLSQS and breast cancer gene transfer was even more efficient with AAV8-ESGLSQS and AAV9-ESGLSQS than with AAV2-ESGLSQS.

## Discussion

Targeted gene transfer has been a major area of research in molecular medicine during the past decade. For adeno-associated viral vectors, both the exploitation of serotypes with their unique transduction profiles [34] as well as the introduction of targeting peptides in the capsid, mainly of AAV2 [23], [24], [25], [26], [27], [32], [33], and DNA shuffling from different serotypes [45], [46], [47] have been explored for targeted gene delivery. AAV8 and AAV9 are particularly attractive for systemic gene delivery because they mediate strong transgene expression and have the ability to cross endothelial barriers in some organs [5], [6], [7]. However, unlike for AAV2, their natural tropism has never been modified by targeting peptides to re-direct or confine gene transfer to certain tissues *in vivo*. Here we explored whether peptides selected for tissue targeting of AAV2 can be inserted at equivalent sites within the capsids of AAV8 and AAV9. We found that such insertion is compatible with capsid assembly of both serotypes and that it indeed modifies the tropism *in vivo*, strongly depending on the peptide inserted and on the serotype of the capsid it was inserted in.

Koerber *et al.* recently described position 590 in the AAV8 capsid as a potential candidate site for peptide insertion and re-targeting AAV8 vectors [40]. However, no potential insertion site has been described for serotype 9 so far. For AAV2, such peptides have mostly been introduced into capsid regions adjacent to N587 and R588. Comparing the capsid protein sequences of AAV2, AAV8 and AAV9, we found differences among amino acids located in subloop 4 including positions 585–595. This may indicate that these regions are also involved in binding to serotype-specific cellular attachment receptors of AAV8 and -9 which is congruent with some binding characteristics that have been described for these serotypes. AAV2 binds to heparan sulfate proteoglycan (HSPG) *via* the basic amino acid residues R484, R487, K532, R585 and R588 located near the peak of the threefold spike. Binding to HSPG has not been reported for AAV8 and AAV9 [48]. For AAV8 and -9, the laminin receptor (lamR) has been identified as a potential cellular receptor, but this receptor may also serve as co-receptor for AAV2 [39]. For AAV8, the interaction with lamR is essential for transduction of cells *in vitro* and it is mediated by capsid domains near the three-fold spike region (593–623) [39]. Recently, terminal *N*-linked galactose has been described as cellular surface glycan that mediates binding of AAV9 to its host cells [49].

Our primary hypothesis was that the insertion of peptide ligands into the capsid sites of AAV8 and AAV9 can alter the tropism of these AAV serotypes *in vivo*. This hypothesis seems to be confirmed by our findings. Peptide insertion into these sites indeed interferes with the transduction pattern of all three investigated serotypes. Of note, each peptide had a distinct influence on tropism in each serotype. Besides the binding characteristics of the inserted peptide, changes in amino acids surrounding the insertion point may also contribute to the modified tropism. This may apply especially for AAV8, because in this serotype the cloning procedure necessitated the insertion of a charged amino acid upstream of the peptide insert.

The insertion of a control peptide (VRRPRFW) into the capsid of AAV2, -8 and -9 resulted in transduction-deficient vectors as expected, since this peptide had not been optimized to mediate transduction into a certain tissue. The strong cationic nature of residues within VRRPRFW might suggest heparin interaction of this capsid-modified AAV, but we have shown that VRRPRFW peptide insertion into the AAV2 capsid domain surrounding R588 results in a heparin binding deficient capsid phenotype [31].

Of note, the tropism the peptides were selected for by screening random AAV2 peptide libraries was not *per se* transferable from one serotype to another. Exceptions were the random peptide control VRRPRFW which conferred ablation of transduction capacity in all serotypes, and the ESGLSQS peptide that resulted in a comparable transduction pattern both in the original structural context AAV2 and AAV9.

Random peptide libraries displayed on AAV capsids can be screened for capsid variants that overcome the natural AAV transduction resistance of certain tissues *in vivo* [32], [33]. One of the major strengths of screening AAV libraries is that it takes into account the unique protein context of the capsid surrounding the targeting ligand.

Yet, despite the fact that the complex capsid structure of AAV8 and AAV9 may be different from AAV2 in the domain we inserted the ligands in, there is some evidence that the use of alternative parental serotype capsids may also provide advantages. In fact, gene delivery to breast tumor tissue by our novel vectors AAV8-ESGLSQS and AAV9-ESGLSQS is superior compared to the AAV2 capsid that has been selected by AAV2 library screening.

The comparable *in vivo* transduction profile of AAV9 and AAV2 displaying ESGLSQS suggests that both vector variants may use the same molecular transduction mechanism presumably by binding to the same receptors or class of receptors. Another pertinent point is that peptide insertion into the threefold spike domain of AAV serotypes 8 and 9 could further facilitate the development of vectors able to escape host-derived neutralizing antibodies as it has been shown for AAV2 vectors [36]. This is especially relevant in view of the fact that there is little prevalence of neutralizing antibodies even to wild type AAV8 and -9 in the general population [11].

AAV2 vectors displaying PRSTSDP were selected for optimized lung transduction. This peptide expanded instead of restricted AAV2 tropism to the lung, because the endogenous AAV2 tropism does not allow for lung transduction and, in turn, AAV2-PRSTSDP vector tropism included but was not limited to the lung [32]. In contrast to the transduction of tumor tissue with vectors displaying the ESGLSQS peptide, the best transduction of lung tissue was observed with the PRSTSDP peptide when it was displayed in AAV2. Transfer of the peptide to the two other serotypes did not result in equally efficient gene delivery to this tissue. This suggests that at least for some peptides the parental serotype that has been used for peptide library screening is crucial for the transduction of the target tissue.

Taken together, we observed both improved target tissue transduction with peptides in AAV8 and AAV9 as opposed to AAV2 on the one hand and optimal transduction only in the original AAV2 context on the other hand. Therefore, we suggest that random peptide libraries displayed on AAV8 and AAV9 should be established and screened *in vivo* to select AAV with optimal specificity and efficiency for the target tissue.
